# Heteroduplex oligonucleotide technology boosts gene knockdown in cardiac and skeletal muscles

**DOI:** 10.1093/nar/gkag007

**Published:** 2026-01-28

**Authors:** Sakino Matsuda-Kawabata, Tetsuya Nagata, Mitsugu Yanagidaira, Kensuke Ihara, Masaki Ohyagi, Hidetoshi Kaburagi, Kie Yoshida-Tanaka, Asuka Sasaki, Aya Abe, Satoe Ebihara, Juri Hasegawa, Nozomi Toide, Masahiro Ohara, Mitsuru Naito, Kanjiro Miyata, Kazuko Toh, Fumika Sakaue, Takanori Yokota

**Affiliations:** Department of Neurology and Neurological Science, Graduate School of Medical and Dental Sciences, Institute of Science Tokyo, 1-5-45 Yushima, Bunkyo-ku, Tokyo 113-8519 Tokyo, Japan; Center for Brain Integration Research, Institute of Science Tokyo, 1-5-45 Yushima, Bunkyo-ku, Tokyo 113-8519 Tokyo, Japan; NucleoTIDE and PepTIDE Drug Discovery Center, Institute of Science Tokyo, 1-5-45 Yushima, Bunkyo-ku, Tokyo 113-8519 Tokyo, Japan; Department of Neurology and Neurological Science, Graduate School of Medical and Dental Sciences, Institute of Science Tokyo, 1-5-45 Yushima, Bunkyo-ku, Tokyo 113-8519 Tokyo, Japan; Center for Brain Integration Research, Institute of Science Tokyo, 1-5-45 Yushima, Bunkyo-ku, Tokyo 113-8519 Tokyo, Japan; NucleoTIDE and PepTIDE Drug Discovery Center, Institute of Science Tokyo, 1-5-45 Yushima, Bunkyo-ku, Tokyo 113-8519 Tokyo, Japan; Department of Neurology and Neurological Science, Graduate School of Medical and Dental Sciences, Institute of Science Tokyo, 1-5-45 Yushima, Bunkyo-ku, Tokyo 113-8519 Tokyo, Japan; Center for Brain Integration Research, Institute of Science Tokyo, 1-5-45 Yushima, Bunkyo-ku, Tokyo 113-8519 Tokyo, Japan; NucleoTIDE and PepTIDE Drug Discovery Center, Institute of Science Tokyo, 1-5-45 Yushima, Bunkyo-ku, Tokyo 113-8519 Tokyo, Japan; Department of Cardiovascular Medicine, Institute of Science Tokyo, 1-5-45 Yushima, Bunkyo-ku, Tokyo 113-8519 Tokyo, Japan; Department of Neurology and Neurological Science, Graduate School of Medical and Dental Sciences, Institute of Science Tokyo, 1-5-45 Yushima, Bunkyo-ku, Tokyo 113-8519 Tokyo, Japan; Center for Brain Integration Research, Institute of Science Tokyo, 1-5-45 Yushima, Bunkyo-ku, Tokyo 113-8519 Tokyo, Japan; Department of Neurology and Neurological Science, Graduate School of Medical and Dental Sciences, Institute of Science Tokyo, 1-5-45 Yushima, Bunkyo-ku, Tokyo 113-8519 Tokyo, Japan; Center for Brain Integration Research, Institute of Science Tokyo, 1-5-45 Yushima, Bunkyo-ku, Tokyo 113-8519 Tokyo, Japan; Department of Neurology and Neurological Science, Graduate School of Medical and Dental Sciences, Institute of Science Tokyo, 1-5-45 Yushima, Bunkyo-ku, Tokyo 113-8519 Tokyo, Japan; Center for Brain Integration Research, Institute of Science Tokyo, 1-5-45 Yushima, Bunkyo-ku, Tokyo 113-8519 Tokyo, Japan; NucleoTIDE and PepTIDE Drug Discovery Center, Institute of Science Tokyo, 1-5-45 Yushima, Bunkyo-ku, Tokyo 113-8519 Tokyo, Japan; Department of Neurology and Neurological Science, Graduate School of Medical and Dental Sciences, Institute of Science Tokyo, 1-5-45 Yushima, Bunkyo-ku, Tokyo 113-8519 Tokyo, Japan; Center for Brain Integration Research, Institute of Science Tokyo, 1-5-45 Yushima, Bunkyo-ku, Tokyo 113-8519 Tokyo, Japan; NucleoTIDE and PepTIDE Drug Discovery Center, Institute of Science Tokyo, 1-5-45 Yushima, Bunkyo-ku, Tokyo 113-8519 Tokyo, Japan; Department of Neurology and Neurological Science, Graduate School of Medical and Dental Sciences, Institute of Science Tokyo, 1-5-45 Yushima, Bunkyo-ku, Tokyo 113-8519 Tokyo, Japan; Center for Brain Integration Research, Institute of Science Tokyo, 1-5-45 Yushima, Bunkyo-ku, Tokyo 113-8519 Tokyo, Japan; NucleoTIDE and PepTIDE Drug Discovery Center, Institute of Science Tokyo, 1-5-45 Yushima, Bunkyo-ku, Tokyo 113-8519 Tokyo, Japan; Department of Neurology and Neurological Science, Graduate School of Medical and Dental Sciences, Institute of Science Tokyo, 1-5-45 Yushima, Bunkyo-ku, Tokyo 113-8519 Tokyo, Japan; Center for Brain Integration Research, Institute of Science Tokyo, 1-5-45 Yushima, Bunkyo-ku, Tokyo 113-8519 Tokyo, Japan; Department of Neurology and Neurological Science, Graduate School of Medical and Dental Sciences, Institute of Science Tokyo, 1-5-45 Yushima, Bunkyo-ku, Tokyo 113-8519 Tokyo, Japan; Center for Brain Integration Research, Institute of Science Tokyo, 1-5-45 Yushima, Bunkyo-ku, Tokyo 113-8519 Tokyo, Japan; Department of Neurology and Neurological Science, Graduate School of Medical and Dental Sciences, Institute of Science Tokyo, 1-5-45 Yushima, Bunkyo-ku, Tokyo 113-8519 Tokyo, Japan; Center for Brain Integration Research, Institute of Science Tokyo, 1-5-45 Yushima, Bunkyo-ku, Tokyo 113-8519 Tokyo, Japan; Department of Neurology and Neurological Science, Graduate School of Medical and Dental Sciences, Institute of Science Tokyo, 1-5-45 Yushima, Bunkyo-ku, Tokyo 113-8519 Tokyo, Japan; Center for Brain Integration Research, Institute of Science Tokyo, 1-5-45 Yushima, Bunkyo-ku, Tokyo 113-8519 Tokyo, Japan; Department of Materials Engineering, Graduate School of Engineering, The University of Tokyo, 7-3-1 Hongo, Bunkyo-ku 113-8656 Tokyo, Japan; Department of Materials Engineering, Graduate School of Engineering, The University of Tokyo, 7-3-1 Hongo, Bunkyo-ku 113-8656 Tokyo, Japan; Department of Neurology and Neurological Science, Graduate School of Medical and Dental Sciences, Institute of Science Tokyo, 1-5-45 Yushima, Bunkyo-ku, Tokyo 113-8519 Tokyo, Japan; Center for Brain Integration Research, Institute of Science Tokyo, 1-5-45 Yushima, Bunkyo-ku, Tokyo 113-8519 Tokyo, Japan; NucleoTIDE and PepTIDE Drug Discovery Center, Institute of Science Tokyo, 1-5-45 Yushima, Bunkyo-ku, Tokyo 113-8519 Tokyo, Japan; Department of Neurology and Neurological Science, Graduate School of Medical and Dental Sciences, Institute of Science Tokyo, 1-5-45 Yushima, Bunkyo-ku, Tokyo 113-8519 Tokyo, Japan; Center for Brain Integration Research, Institute of Science Tokyo, 1-5-45 Yushima, Bunkyo-ku, Tokyo 113-8519 Tokyo, Japan; NucleoTIDE and PepTIDE Drug Discovery Center, Institute of Science Tokyo, 1-5-45 Yushima, Bunkyo-ku, Tokyo 113-8519 Tokyo, Japan; Department of Neurology and Neurological Science, Graduate School of Medical and Dental Sciences, Institute of Science Tokyo, 1-5-45 Yushima, Bunkyo-ku, Tokyo 113-8519 Tokyo, Japan; Center for Brain Integration Research, Institute of Science Tokyo, 1-5-45 Yushima, Bunkyo-ku, Tokyo 113-8519 Tokyo, Japan; NucleoTIDE and PepTIDE Drug Discovery Center, Institute of Science Tokyo, 1-5-45 Yushima, Bunkyo-ku, Tokyo 113-8519 Tokyo, Japan

## Abstract

Recent advances in gapmer antisense oligonucleotides (ASOs) have led to the regulatory approval of ASO therapeutics that target the liver and central nervous system. Efficient delivery of gapmer ASOs to muscle tissue will further broaden their therapeutic applications. Here, we evaluated the knockdown activity of lipid-conjugated DNA/RNA heteroduplex oligonucleotides (HDOs) in muscle tissue. Cholesterol-conjugated HDO (Chol-HDO) exhibited effective gene suppression in both cardiac and skeletal muscles, outperforming unconjugated ASO and cholesterol-conjugated ASO (Chol-ASO). Chol-HDO and Chol-ASO showed greater binding to low-density lipoprotein (LDL); however, while Chol-ASO also exhibited enhanced binding to other serum proteins, Chol-HDO displayed weaker nonspecific interactions. Mechanistically, Chol-HDO enhanced cellular uptake through an LDL receptor-mediated mechanism. Moreover, subcellular distribution analysis revealed that the duplex structure of Chol-HDO promoted efficient nuclear delivery. Regarding safety, intravenous injection of Chol-ASO induced thrombocytopenia, whereas Chol-HDO mitigated this effect at the same dose. *Ex vivo* platelet activation assays confirmed that platelet activation was significantly more suppressed by Chol-HDO treatment than by Chol-ASO. Taken together, these results underscore the therapeutic potential of HDOs for safe and effective knockdown in muscle tissue, and provide a novel platform for the clinical development of gapmer ASO therapy for muscular disorders.

## Graphical Abstract

**Graphical Abstract:**
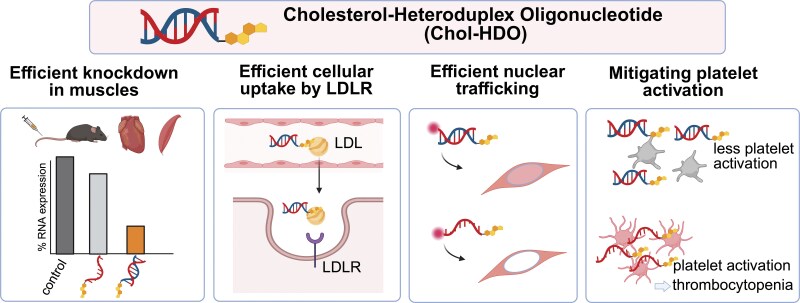

## Introduction

Antisense oligonucleotides (ASOs) are single-stranded nucleic acids consisting of 15–25 nucleotides, which are designed to hybridize with their complementary target RNA sequence to modulate gene expression. ASOs represent a burgeoning field in drug development, offering therapeutic strategies for a variety of diseases that were traditionally considered undruggable via conventional small-molecule methods or small-molecule inhibitors [1–6]. Although disease-modifying therapeutics are limited for hereditary neurodegenerative and muscle disorders, the advent of ASO-based therapies for spinal muscular atrophy [1], Duchenne muscular dystrophy (DMD) [2–4], familial amyloid polyneuropathy [5], and amyotrophic lateral sclerosis [6] portends a hopeful future for ASOs in medicine.

Phosphorothioate (PS)-modified ASOs can be administered to cardiac and skeletal muscles via systemic injections, such as subcutaneous (SC) or intravenous (IV) injection [7]. However, unlike the sinusoidal capillary structure of the liver and the fenestrated endothelium of the kidney, the continuous endothelium of muscles represents a major barrier to the efficient delivery of macromolecular drugs [8]. Thus, while PS-ASOs can be effectively distributed to the cardiac and skeletal muscles, the dose needed to induce antisense effects is markedly higher than that required to produce similar effects in the liver.

Exon-skipping therapy with phosphorodiamidate morpholino oligomer has been developed and approved for the treatment of DMD; however, its disease-modifying effects are inadequate. Moreover, a clinical trial of a gapmer ASO targeting DMPK (Ionis DMPK-2.5Rx) for myotonic dystrophy type 1 (DM1) treatment was terminated because of insufficient drug delivery to muscle tissue and poor efficacy [9]. Many other genetic muscle disorders have been identified as candidates for ASO-based treatment [10–13]. Therefore, developing methods to improve ASO delivery to muscles will markedly enhance their therapeutic efficacy and provide treatment options for patients with these diseases.

Previous studies have demonstrated that lipid-conjugated ASOs, including those conjugated to palmitate, tocopherol, and cholesterol (Palm-ASO, Toc-ASO, and Chol-ASO), enhanced gene knockdown in muscles compared with unconjugated ASO [14, 15]. Palm-ASO exhibits high affinity for albumin and facilitates transcytosis across endothelial cells through caveolin1 [16], which is expressed in endothelial cells and various other tissues, but not in muscle fibers [17, 18]. However, after transport across endothelial cells, a large portion of Palm-ASO is sequestered in the interstitial space and cleared without the functional cellular uptake [16]. Moreover, following cellular uptake, the amount of ASO and lipid-conjugated ASO (lipid-ASO) escaping from endosomes is limited [19, 20]. Therefore, to enhance the therapeutic efficacy in muscle, improvements in tissue delivery, cellular uptake, and endosomal escape are necessary.

Furthermore, although cholesterol conjugation enhanced ASO potency more effectively than palmitate following IV administration, Chol-ASO has exhibited lethal toxicity after IV injection [14]. In addition, PS-ASO activates platelets via the glycoprotein VI (GPVI) pathway [21–23], and cholesterol conjugation facilitates this process, resulting in thrombocytopenia [24, 25].

We developed a DNA/RNA heteroduplex oligonucleotide (HDO) that exhibits highly efficient activity compared with ASOs [26–33]. HDO is composed of a gapmer ASO duplexed with a complementary strand that is conjugated to a delivery ligand, such as tocopherol (Toc-HDO) or cholesterol [cholesterol-conjugated HDO (Chol-HDO)]. In our previous study, a high-dose regimen of Chol-HDO (50 mg/kg once a week for 4 weeks) resulted in significant gene suppression in skeletal muscle [28]; however, the efficacy, dose, and dosing regimens of Toc-HDO and Chol-HDO for the cardiac and skeletal muscles, including the diaphragm, have not been optimized. In addition, the mechanism underlying the efficient suppression of the target genes in muscle and the tolerability of lipid-HDOs remain unclear, particularly when compared with lipid-ASOs.

In this study, we demonstrate that Chol-HDO exhibits a highly potent knockdown effect in both cardiac and skeletal muscles, surpassing that observed with unconjugated ASO and lipid-ASO, while reducing the risk of thrombocytopenia. We also discovered that efficient tissue delivery and nuclear trafficking contribute to HDO potency.

## Materials and methods

### Oligonucleotide synthesis

Chemically modified oligonucleotides were synthesized by GeneDesign, Inc. (Osaka, Japan). The constructs are listed in Supplementary Table S1. To generate HDOs, we hybridized equimolar concentrations of the ASOs and complementary strands by denaturing them at 95°C for 5 min. Subsequently, they were slowly cooled to 37°C to facilitate annealing.

### Animals

Six-week-old wild-type (WT) male C57BL/6J mice were obtained from Oriental Yeast (Tokyo, Japan) and maintained under a 12 h light/12 h dark cycle in a pathogen-free animal facility with ad libitum access to food and water. All experiments were approved by the Institutional Animal Care and Use Committee of the Institute of Science Tokyo and were conducted according to the ethical and safety guidelines for animal experiments of the Tokyo Medical and Dental University and the Institute of Science Tokyo (approval numbers A2022-085A and A2023-144C2). Low-density lipoprotein (LDL) receptor knockout mice (B6;129S7-*Ldlr^tm1Her^*/J; Strain 002077), caveolae protein 1 knockout mice (B6.Cg-*Cav1^tm1Mls^*/J; Strain 007083), and macrophage scavenger receptor 1 knockout mice (B6.Cg-*Msr1^tm1Csk^*/J; Strain 006096) were purchased from the Jackson Laboratory (Bar Harbor, ME, USA).

### Administration of ASOs and HDOs

Phosphate-buffered saline (PBS) or the oligonucleotides were injected intravenously through the tail vein or subcutaneously based on the mouse body weight. For convenient comparison of oligonucleotides with differing molecular weights, the administered dose of unconjugated HDO, lipid-ASO, and lipid-HDO was based on the unconjugated ASO quantity. The oligonucleotides were formulated in PBS prior to administration.

### Quantitative reverse transcriptase-polymerase chain reaction

Total RNA was extracted from muscle tissues using the MagNA Pure 96 system (Roche Diagnostics, Basel, Switzerland) following the manufacturer’s instructions. DNase-treated RNA (0.3 µg) was reverse-transcribed using PrimeScript™ RT Master Mix (Takara Bio Inc., Shiga, Japan; Cat. RR036A). Quantitative reverse transcriptase-polymerase chain reaction (qRT-PCR) was performed using the LightCycler 480 Real-Time PCR instrument (Roche Diagnostics, Basel, Switzerland). Primers and probes specific to the mouse genes encoding for actin beta (*Actb)*, glyceraldehyde 3-phosphate dehydrogenase *(Gapdh)*, metastasis-associated lung adenocarcinoma transcript 1 (*Malat1)*, dystrophia myotonica protein kinase *(Dmpk)*, and scavenger receptor class B member 1 (*Scarb1*) were obtained from Thermo Fisher Scientific (Waltham, MA, USA) and synthesized at FASMAC Co., Ltd. (Atsugi, Japan). The relative gene expression values are presented as means ± standard error of the mean (SEM) using the 2^−ΔΔCt^ method, in which ΔCt represents the difference between the mean cycle threshold (Ct) values of duplicate measurements for target gene expression and *Actb* or *Gapdh* as an internal control. ΔΔCt represents the difference between the ΔCt values of the treated and vehicle control groups.

### Immunoblotting

For immunoblotting of DMPK, muscle samples were homogenized on ice in an NP-40 lysis buffer [1% NP-40, 50 mM Tris–HCl, pH 7.5, 150 mM NaCl, 1 mM ethylenediaminetetraacetic acid (EDTA), 1% sodium dodecyl sulphate (SDS), Complete Ultra protease inhibitor Tablet; Roche Life Science, Basel, Switzerland; Cat. 5892953001] using a Potter–Elvehjem homogenizer. The lysates were centrifuged for 10 min at 14 000 × *g* at 4°C, and the supernatants were subjected to bicinchoninic acid assay (Thermo Fisher Scientific; Cat. 23225) to quantify protein concentration. Total protein (1.5 µg for Heart and 7.5 μg for Quadriceps) was subjected to 10% polyacrylamide gel electrophoresis and transferred to polyvinylidene fluoride membranes (Bio-Rad Laboratories, Hercules, CA, USA). The membranes were blocked with 5% skim milk for 1 h at room temperature (RT; 25°C) and incubated with the following primary antibodies overnight at 4°C: anti-mouse DMPK (Thermo Fisher Scientific, Waltham, MA, USA; Cat. 34-9900; 1:200) and anti-mouse GAPDH (Thermo Fisher Scientific, Waltham, MA, USA; Cat. MA5-15738; 1:5000). The membranes were incubated with the following secondary antibodies for 1 h at RT: goat anti-rabbit horseradish peroxidase-conjugated antibody (Jackson ImmunoResearch Laboratories Inc., West Grove, PA, USA; Cat. 111-035-003) and goat anti-mouse horseradish peroxidase-conjugated antibody (Jackson ImmunoResearch Laboratories Inc., West Grove, PA, USA; Cat. 115-035-003), at a dilution of 1:3333 and 1:10 000, respectively. SuperSignal West Dura Extended Duration Substrate (Thermo Fisher Scientific, Waltham, MA, USA; Cat. 34075) and the ChemiDoc MP Imaging System (Bio-Rad Laboratories, Hercules, CA, USA) were used to detect the DMPK and GAPDH bands. The band intensities were quantified using ImageJ (US National Institutes of Health, version 1.53).

For immunoblotting of LDLR, C2C12 cells were lysed in NP-40 lysis buffer. Primary antibodies included a polyclonal anti-LDLR antibody (Proteintech Group, Inc., Rosemont, IL, USA; Cat. 10785-1-AP; 1:1000) and an anti-mouse GAPDH antibody (Thermo Fisher Scientific, Waltham, MA, USA; Cat. 34-9900; 1:5000).

### Immunohistochemical analysis using anti-PS antibody

Muscle samples were fixed in 4% paraformaldehyde and embedded in paraffin. The slides were deparaffinized in xylene and pretreated with proteinase K (Dako, Glostrup, Denmark; Cat. S302080-2) at RT for 5 min for antigen retrieval. The slides were then incubated with BLOX ALL (Vector Laboratories, Newark, CA, USA; Cat. SP-6000) for 10 min to inactivate endogenous peroxidase and subsequently treated with Background Buster (Innovex Biosciences, Richmond, CA, USA; Cat. NB306-50) for 30 min. The slides were incubated for 1 h at RT with polyclonal rabbit anti-PS antibody [34], generously provided by Ionis Pharmaceuticals, diluted at 1:4000 in 10% normal goat serum. The slide was rinsed three times in PBS and incubated with a secondary goat anti-rabbit horseradish peroxidase-conjugated antibody (Jackson ImmunoResearch Laboratories, West Grove, PA, USA, Inc.; Cat. 111-035-003; diluted at 1:200) for 30 min at RT. The slides were developed using 3,3′-diaminobenzidine (DAB) (Thermo Fisher Scientific, Waltham, MA, USA; Cat. 34065).

### SplintR quantitative polymerase chain reaction assay

SplintR qPCR was performed to quantify ASO concentrations in muscle tissues as previously described [35], with minor modifications. Muscle tissues were homogenized in 30 µl/mg of Radioimmunoprecipitation assay (RIPA) buffer (50 mM Tris–HCl, pH 7.5; 150 mM NaCl; 1% NP-40; 0.5% sodium deoxycholate; 0.1% SDS; 5 mM EDTA; 1 mM Ethylene glycol-bis(β-aminoethyl ether)-N,N,N′,N′-tetraacetic acid (EGTA)) using a TissueLyser II (Qiagen, Waltham, MA, USA). Samples were incubated on ice for 1 h with vortexing every 10 min and then centrifuged at 16 000 × *g* for 30 min at 4°C. Supernatants were treated with RNase Cocktail (Thermo Fisher Scientific, Waltham, MA, USA; Cat. AM2286) at 37°C for 30 min to cleave the complementary RNA strand of the HDO. Two microliters of each sample were mixed with 2 µl of 10 × SplintR Ligase Reaction Buffer (New England Biolabs, Ipswich, MA, USA; Cat. M0375S), 2 µl each of probe A (100 nM) and probe B (100 nM), and nuclease-free water to a final volume of 12 µl. The mixtures were denatured at 95°C for 5 min and cooled to 37°C (0.1°C/s) for hybridization. Eight microliters of diluted SplintR ligase (2.5 U/reaction; New England Biolabs, Ipswich, MA, USA; Cat. M0375S) were then added, followed by incubation at 37°C for 30 min and heat inactivation at 65°C for 20 min. Next, 1 µl of the ligation product was used for qPCR in 10 µl of a reaction containing LightCycler 480 Probes Master (Roche Diagnostics, Basel, Switzerland; Cat. 04887301001), forward and reverse primers (500 nM each), and a double-quenched probe (250 nM). Primer and probe sequences are provided in Supplementary Table S2.

### RNAscope *in situ* hybridization

The expression of *Dmpk* in tissues was detected using the RNAscope^®^ 2.5 HD Brown Chromogenic Reagent Kit based on the manufacturer’s instructions (Advanced Cell Diagnostics, Newark, CA, USA; Cat. 322300). Muscle samples were fixed in 4% paraformaldehyde and embedded in paraffin. The samples were cut into 5 µm sections and mounted on SuperFrost Plus slides (Thermo Fisher Scientific, Waltham, MA, USA). The slides were baked in an oven for 1 h at 60°C, incubated twice in xylene for 5 min, and rinsed in 100% ethanol twice for 1 min. The sections were then incubated in hydrogen peroxide for 10 min at RT, rinsed with distilled water, and boiled in 1× RNAscope Target Retrieval Buffer for 15 min. The slides were rinsed in distilled water, transferred to 100% ethanol for 5 min, and air-dried. The tissues were subjected to protease plus treatment and incubated in an oven for 30 min at 40°C. They were rinsed in distilled water twice for 2 min and incubated with Dmpk-specific probes (Advanced Cell Diagnostics, Newark, CA, USA; Cat. 530741) at 40°C for 2 h. The target probe signal was further amplified based on the manufacturer’s instructions. Finally, the sections were developed using DAB (Thermo Fisher Scientific, Waltham, MA, USA; Cat. 34065).

### Fluorescence polarization

The binding affinities of ASOs and HDOs toward plasma proteins were analyzed using fluorescence polarization (FP), as described previously [36]. Mouse serum albumin was purchased from Sigma–Aldrich (St. Louis, MO, USA; Cat. A3139). Mouse transferrin was purchased from Jackson ImmunoResearch Laboratories, Inc. (West Grove, PA, USA; Cat. 015-000-050). Mouse Immunoglobulin G was purchased from Wako Pure Chemical Corporation (Osaka, Japan, 140-09511). Mouse histidine-rich glycoprotein (Cat. 1905-HP-025) and mouse glycoprotein VI recombinant protein (Cat. 6758-GP-050) were purchased from R&D Systems (Minneapolis, MN, USA). LDL was isolated from mouse serum using ultracentrifugation as previously described [37]. Unconjugated ASOs and HDOs were labeled at the 5′-terminus with Alexa 647, while 5′-Chol-ASO was labeled at the 3′ terminus. Alexa 647-labeled ASOs or HDOs were added at a final concentration of 2 nM to protein solutions of varying concentrations in 1 × DPBS in flat-bottom 384-well plates (Greiner Bio-One, Kremsmünster, Austria; Cat. 781096), which were coated with Sigmacote (Sigma–Aldrich, St. Louis, MO, USA; Cat. SL2). The plates were incubated at RT for at least 30 min, and polarization was measured using Infinite M1000 PRO (Tecan, Männedorf, Switzerland). The excitation and emission wavelengths were 635 and 675 nm, respectively.

### Cell culture and transfection

C2C12 cells were seeded on collagen-coated 48-well plates in high-glucose Dulbecco’s modified Eagle’s media (DMEM; high glucose; Wako Pure Chemical Corporation, Osaka, Japan; Cat. 04429765) containing 10% fetal bovine serum (FBS) and 1% penicillin–streptomycin. They were cultured until they reached 80%–90% confluency. Differentiation was induced by replacing the medium with differentiation medium (DMEM-high glucose supplemented with 2% horse serum and 1% penicillin–streptomycin). For the LDLR overexpression study, the cells were transfected with mouse *Ldlr* plasmid (OriGene Technologies, Inc., Rockville, MD, USA) at a concentration of 300 ng/well for 48 h using Lipofectamine 3000 (Thermo Fisher Scientific, Waltham, MA, USA). The cells were then treated with ASOs and HDOs via gymnosis in DMEM-high glucose supplemented with 1% mouse serum. After 24 h, RNA was extracted using the FastGene RNA Basic Kit (Nippon Genetics, Tokyo, Japan; Cat. FG-80250) according to the manufacturer’s protocol.

### Subcellular distribution analysis of ASO, HDO, Chol-ASO, and Chol-HDO

Alexa 647 was conjugated to the 5′-terminus of the ASO, 5′-terminus of the ASO strand of HDO and Chol-HDO, and 3′ terminus of Chol-ASO. C2C12 cells were seeded onto collagen-coated eight-well culture slides (SPL Life Sciences, Pocheon, South Korea; Cat. SPL-30108) and differentiation was induced using differentiation medium. LDLR overexpression and gymnosis of ASO, 5′-Chol-ASO, HDO, and Chol-HDO with 1% mouse serum were performed using the aforementioned methods. The medium was replaced 6 h after gymnosis. The slides were fixed with 4% paraformaldehyde for 30 min, and the nuclei were stained with DAPI. Images were captured using the Nikon A1R laser scanning confocal microscope (Nikon, Tokyo, Japan). Alexa 647 signals were excited using a 646 nm laser and detected with a 700 nm emission filter.

### Platelet activation assay

Whole mouse blood was collected into tubes containing 3.2% sodium citrate and centrifuged at 200 × *g* for 10 min at RT to obtain platelet-rich plasma (PRP). For the calcium influx assay, purified platelets were incubated at 37°C for 30 min in the presence of Fluo-8 AM (5 µM; AAT Bioquest, Pleasanton, CA, USA; Cat. 21081), 1.25 mM probenecid (Wako Pure Chemical Corporation, Osaka, Japan; Cat. 28920–32), and 0.05% Pluronic F-127 (Biotium, Fremont, CA, USA; Cat. 59000). The platelets were then collected by centrifugation at 400 × *g* for 10 min and resuspended in HBSS containing 2 mM Ca^2+^ and 1.25 mM probenecid. Subsequently, PBS, ASO, Chol-ASO, or Chol-HDO was added, and the fluorescence intensity was monitored for 30 min using Infinite M1000 PRO (Tecan, Männedorf, Switzerland).

### Statistical analysis

Statistical analysis was performed using GraphPad Prism 10 software and Microsoft Excel. All experimental data are expressed as mean ± SEM. Differences between more than two groups were compared using the one-way analysis of variance, followed by Tukey’s or Dunnett’s multiple-comparisons test. Statistical differences between the two groups were determined using the Student’s two-tailed *t*-test. The significance criterion was set at *P* <.05.

## Results

### Activity of lipid-HDO targeting *Malat1* in cardiac and skeletal muscles of mice

**Figure 1.**
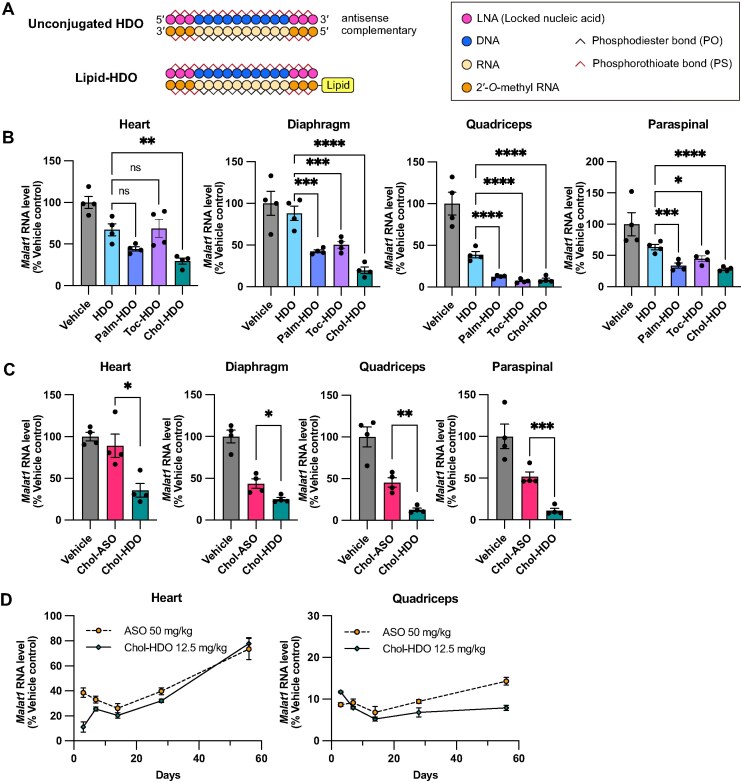
Knockdown activity of lipid-HDOs and Chol-ASO targeting *Malat1* in cardiac and skeletal muscles of mice. **(A)** Structures of unconjugated and lipid-conjugated HDO. **(B)**  *Malat1* RNA levels in the heart, diaphragm, quadriceps, or paraspinal muscles following a single intravenous injection of PBS (Vehicle control), unconjugated HDO, Palm-HDO, Toc-HDO, or Chol-HDO targeting *Malat1* at a dose of 5 mg/kg. *n* = 4 per group. Muscles were collected 3 days after dosing. Statistical analysis using one-way analysis of variance followed by Dunnett’s test was performed. **(C)** Head-to-head comparison of *Malat1* knockdown between Chol-ASO and Chol-HDO 3 days after an intravenous injection of 12.5 mg/kg. *n* = 4 per group. Statistical significance was assessed by the Student’s *t*-test. **(D)** Time course of *Malat1* RNA suppression in the heart, diaphragm, quadriceps, or paraspinal muscles on days 3, 7, 14, 28, or 56 following a single intravenous injection of 50 mg/kg of unconjugated ASO or 12.5 mg/kg of Chol-HDO. The doses represent the mass of the unconjugated ASO moiety. All data are presented as the mean ± SEM. *P <.05; **P <.01; ***P <.001; ****P <.0001; ns: not significant.

We examined the knockdown activity of lipid-conjugated DNA/RNA heteroduplex oligonucleotides (lipid-HDOs) targeting *Malat1* in the muscle. *Malat1* is a long noncoding RNA that is ubiquitously expressed in mouse tissues. Because conjugation with cholesterol, tocopherol, and palmitate was shown to enhance the activity of single-stranded ASOs in muscle [14–16], these lipids were attached to the 5′ end of the complementary strand to produce lipid-HDOs (Fig. 1A). Three days after IV injection of the lipid-HDOs or unconjugated HDO at a dose of 5 mg/kg, *Malat1* RNA expression was quantified in cardiac and skeletal muscles using qRT-PCR. Among the tested HDOs, the Chol-HDO exhibited the highest knockdown activity in both cardiac and skeletal muscles (Fig. 1B).

To determine how the chemical structure influences HDO activity, we compared the HDOs with cholesterol conjugated at the 5′- or 3′ end of the complementary strand (5′-Chol-HDO [Chol-HDO] or 3′-Chol-HDO). Neither unconjugated HDO nor 3′ Chol-HDO enhanced the activity of the unconjugated ASO, whereas 5′-Chol-HDO showed superior activity (Supplementary Fig. S1). This suggests that the double-stranded structure alone is not sufficient for effective knockdown and that the specific cholesterol attachment position is also an important determinant of knockdown activity. This result is consistent with our previous study indicating that the ligand position on HDOs affects their activity in the central nervous system following systemic administration [28].

### Direct comparison of Chol-ASO and Chol-HDO

Lipid-ASOs effectively enhance tissue delivery compared with unconjugated ASOs [14–16]. To directly compare the activity of Chol-HDO and Chol-ASO, we administered them at an equivalent ASO dose of 12.5 mg/kg. Three days following the IV injection, Chol-HDO exhibited a superior knockdown activity in all muscle tissues tested compared with Chol-ASO (Fig. 1C), suggesting that the HDO platform offers advantages in cholesterol-based drug delivery.

### Dose–response study of Chol-HDO

To determine the dose–response relationships, ASO, Toc-HDO, and Chol-HDO were administered via IV injection at 5, 12.5, 25, or 50 mg/kg (based on ASO content) (Supplementary Fig. S2). Considering that the molecular weight of HDO is approximately twice that of ASO, the actual oligonucleotide dose of HDO based on weight was approximately twofold higher. Nonetheless, Chol-HDO at an ASO-equimolar dose of 12.5 mg/kg (∼25 mg/kg total oligonucleotide weight) still demonstrated superior knockdown compared to 25 mg/kg of unconjugated ASO in both cardiac and skeletal muscles (Supplementary Fig. S2A). Indeed, the ED_90_ value of Chol-HDO (ASO-equimolar dose) was at least 2.7- to 6.1-fold lower than that of unconjugated ASO (Supplementary Fig. S2B). The results indicate that Chol-HDO achieves higher efficacy and potency than unconjugated ASO, even when administered on an equivalent weight basis. Notably, the scrambled ASO and scrambled Chol-HDO did not show any significant knockdown effects, indicating that the knockdown observed in the present study was sequence specific [38].

### Chol-HDO exhibits long-lasting activity

We examined the duration of the knockdown activity in the heart and quadriceps following a single administration of 50 mg/kg ASO and Chol-HDO at a molar dose equivalent to 12.5 mg/kg of ASO. Despite a fourfold difference in dose, a trend in reduction was similar for 56 days in the heart after administration (Fig. 1D). In the quadriceps, the reduction of RNA by Chol-HDO was similar to that achieved by ASO during the first 14 days following administration; however, Chol-HDO enhanced RNA reduction on days 28 and 56 after administration (Fig. 1D). To further examine the long-term effects on RNA levels, the mice were intravenously administered Chol-HDO at the same molar dose as 50 mg/kg ASO. Even at 252 days after administration, a >70% reduction in RNA levels in the quadriceps and paraspinal muscles and ∼50% in the diaphragm was observed (Supplementary Fig. S3). Furthermore, RNA reduction was observed up to 168 days following administration in the heart, although the effect decreased more rapidly in the heart than in the skeletal muscles (Supplementary Fig. S3).

### Effect of the administration route on lipid-HDO activity

The administration routes were compared with respect to lipid-HDO potency. IV administration of Toc-HDO showed similar potency to SC delivery in most tissues, except in the heart, in which IV injection was superior (Supplementary Fig. S4A). Similarly, SC administration of Chol-HDO achieved effective knockdown; however, IV administration resulted in greater potency in the heart and paraspinal muscles (Supplementary Fig. S4B). These results align with those of a previous report showing that SC injection of cholesterol- or tocopherol-conjugated ASOs yields lower muscle potency than IV injection [14].

### Activity of *Dmpk* and *Scarb1* Chol-HDO in mouse cardiac and skeletal muscles

**Figure 2.**
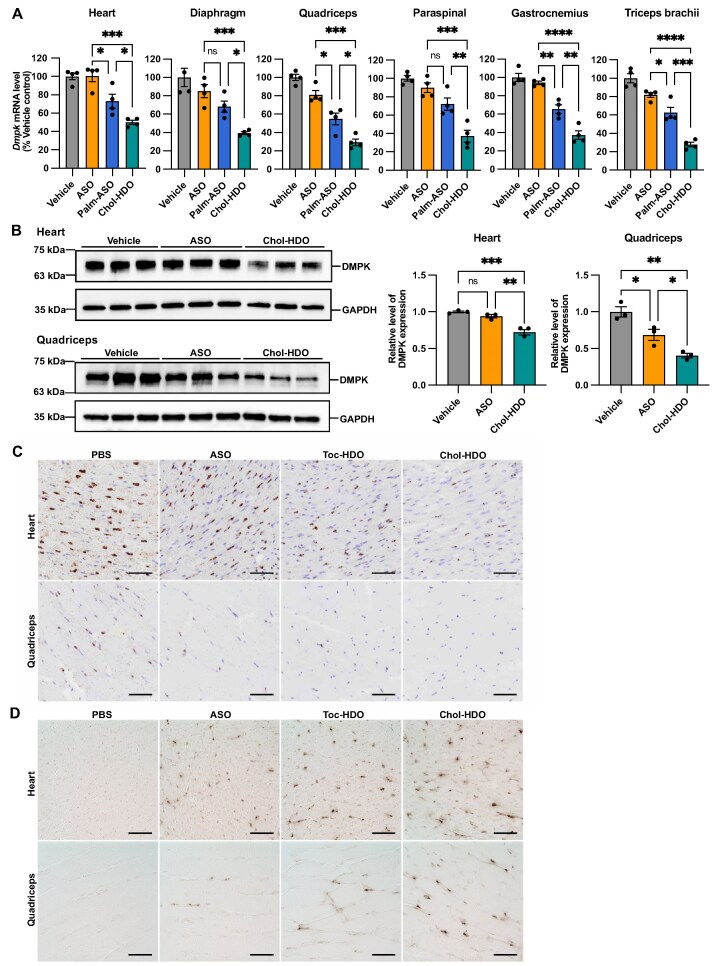
Knockdown activity of unconjugated ASO, Palm-ASO, or Chol-HDO targeting *Dmpk* in the cardiac and skeletal muscles of mice. **(A)**  *Dmpk* mRNA expression in the heart, diaphragm, quadriceps, paraspinal muscles, gastrocnemius, and triceps brachii 3 days after a single intravenous injection of vehicle control, unconjugated ASO, Palm-ASO, or Chol-HDO at a dose of 12.5 mg/kg. *n* = 4 per group. **(B)** Immunoblotting and densitometric analysis (*n* = 3 per group) of the heart and quadriceps (1.5 and 7.5 µg of total protein, respectively) in mice treated with vehicle control, 12.5 mg/kg ASO-, or Chol-HDO. **(C)**  *In situ* hybridization using the *Dmpk* probe in the heart and quadriceps 3 days after a single injection of PBS, 50 mg/kg of unconjugated ASO, Toc-HDO, or Chol-HDO at equimolar doses to the unconjugated ASO; scale bar: 50 μm. **(D)** Immunohistochemistry staining with an anti-PS antibody in the heart and quadriceps harvested from mice 3 days after injection of 25 mg/kg of unconjugated ASO, Toc-HDO, or Chol-HDO targeting *Dmpk* or PBS alone. Scale bar: 50 μm. The doses represent the mass of the unconjugated ASO moiety. All graphs represent the mean ± SEM. Statistical analysis was performed using one-way analysis of variance followed by Tukey’s multiple-comparisons test. *P <.05; **P <.01; ***P <.001; ****P <.0001; ns: not significant.

The expansion of CTG repeats in the 3′ untranslated regions (UTR) of human *DMPK* results in myotonic dystrophy type 1 (DM1), the most common type of muscular dystrophy in adults. The symptoms include muscle weakness, myotonia, and other systemic symptoms. Downregulation of the *DMPK* gene is considered a promising therapeutic strategy for treating DM1 [39, 40]. We examined the activity of ASO, Chol-ASO, and Chol-HDO administered at 25 mg/kg, which targeted non-CUG sequences in the 3′ UTR of the mouse *Dmpk* gene. ASO and Chol-HDO were well-tolerated; however, all the four mice treated with Chol-ASO exhibited hemoptysis and died within 24 h (Supplementary Fig. S5). This observation was consistent with a previous report that Chol-ASO targeting *Dmpk* is lethal [14]. In the same study, Palm-ASO was considered a tolerable alternative. Therefore, we compared ASO, Palm-ASO, and Chol-HDO targeting *Dmpk*. The mice were euthanized 72 h after injection, and the muscle tissues were collected to assess *Dmpk* mRNA knockdown by qRT-PCR. The results indicated that Chol-HDO was more active than Palm-ASO in knockdown *Dmpk* mRNA (Fig. 2A).

A dose–response study was conducted for ASO, Toc-HDO, and Chol-HDO targeting *Dmpk*. All the three oligonucleotides reduced *Dmpk* mRNA levels in a dose-dependent manner in all the muscle tissues examined. While ED_50_ values of ASO were >50 mg/kg in all muscles, except the diaphragm, Toc-HDO and Chol-HDO exhibited >1.5- to 2.7-fold or 3.0- to 9.5-fold enhancement in potency and improved efficacy compared with ASO (Supplementary Fig. S6). Similar to the *Malat1* study, we evaluated ASO and Chol-HDO with a scrambled sequence of *Dmpk*-targeting ASO, which did not exert a knockdown effect on *Dmpk* expression.

The reduction in expression of DMPK protein was assessed by immunoblotting. After administering Chol-HDO at the same molar dose as 12.5 mg/kg ASO, a significant reduction in DMPK protein was observed in the heart and quadriceps compared with that observed for ASO (Fig. 2B).

To further determine the versatility of HDO technology, we examined the activity of Toc-HDO and Chol-HDO targeting another gene. *Scarb1*-targeting Toc-HDO and Chol-HDO showed a significantly greater knockdown effect than the equimolar unconjugated ASO (Supplementary Fig. S7).

### Distribution of the reduction in target RNA levels

*In situ* RNA hybridization analysis was performed to evaluate the spatial distribution of mRNA reduction. The mice received IV injections of PBS or the equivalent molar amount of ASO, Toc-HDO, and Chol-HDO targeting *Dmpk*. The mice were euthanized 72 h post-treatment. *Dmpk* signal intensity was recorded in the heart and quadriceps using RNAscope. A higher *Dmpk* signal intensity was observed in the heart compared with that in the quadriceps (Fig. 2C). ASO targeting *Dmpk* reduced the corresponding mRNA levels in both tissues. In contrast, a marked reduction in *Dmpk* expression was observed in both tissues obtained from mice treated with Toc-HDO or Chol-HDO, which was consistent with the qRT-PCR results (Fig. 2A). In particular, Chol-HDO showed a greater reduction in *Dmpk* mRNA levels in both tissues compared with Toc-HDO.

### Muscle-tissue distribution of ASO and lipid-HDOs

A pan anti-PS polyclonal antibody [34] was used for PS staining to determine a potential correlation between ASO distribution and relative potency. Mice were intravenously administered 25 mg/kg of unconjugated ASO, equivalent molar amounts of Toc-HDO and Chol-HDO, or PBS alone, and euthanized 72 h later (Fig. 2D). Immunohistochemical staining showed a wide distribution throughout the heart and quadriceps. As predicted by gene knockdown, the PS signal intensity was highest in the heart and quadriceps of the mice treated with Chol-HDO. In contrast, low signal intensity was observed in both tissues of the mice treated with ASO. To further validate the difference in ASO and Chol-HDO delivery to muscle tissue, we performed SplintR qPCR to quantify ASO concentrations in muscle samples. Mice were intravenously injected with 12.5 mg/kg unconjugated ASO, an equimolar dose of Chol-HDO, or PBS alone. ASO concentrations in the heart and quadriceps of the Chol-HDO–treated mice were 8.4- and 2.9-fold higher, respectively, than those in the ASO-treated mice (Supplementary Fig. S8). These results suggest that improved delivery to muscle tissues contributes to the enhanced knockdown efficiency of lipid-HDOs.

### Affinity of lipid-conjugated HDOs for mouse plasma protein

**Figure 3.**
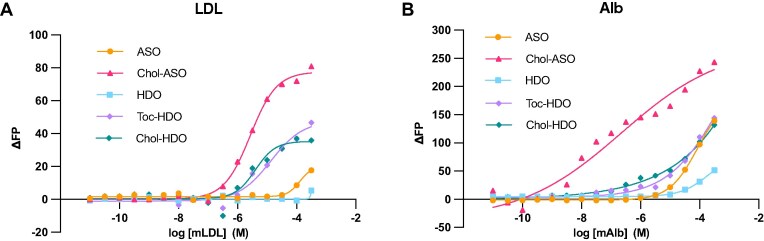
Interaction of oligonucleotides with mouse LDL or albumin. **(A, B)** Binding curves of unconjugated ASO, Chol-ASO, unconjugated HDO, Toc-HDO, and Chol-HDO to mouse LDL **(A)** or albumin **(B)**, as determined by fluorescence polarization (FP).

To elucidate the mechanism underlying the improved delivery of Chol-HDO to muscle tissues, we examined its interaction with putative binding proteins. Oligonucleotides can bind to plasma proteins, and this interaction contributes to delivery efficiency [8, 41]. Specifically, albumin and serum lipoprotein are involved in the oligonucleotide delivery [16, 26, 42]. Therefore, we determined the binding affinities of ASO, Chol-ASO, HDO, Toc-HDO, and Chol-HDO for mouse LDL and albumin by FP. Toc-HDO, Chol-HDO, and Chol-ASO exhibited significantly enhanced binding affinity for LDL, whereas the unconjugated ASO or unconjugated HDO showed minimal binding (Fig. 3A). In contrast, Chol-HDO exhibited only a modest increase in albumin binding, whereas Chol-ASO showed a markedly higher affinity compared with unconjugated ASO or HDO (Fig. 3B). We also assessed the binding to other serum proteins, including mouse transferrin (TF), Immunoglobulin G (IgG), and histidine-rich glycoprotein (HRG) (Supplementary Fig. S9). Chol-ASO showed significantly enhanced binding affinity for IgG and TF, whereas lipid-HDOs exhibited slightly increased or comparable affinity for these proteins. Unconjugated HDO demonstrated a reduced binding affinity to these serum proteins compared with unconjugated ASO. Binding to HRG was nearly identical for all of the tested molecules. These results suggest that despite sharing cholesterol conjugation, Chol-ASO and Chol-HDO differ in their protein binding profiles.

### *In vitro* activity of lipid-conjugated ASO and HDO with co-administration of mouse serum

**Figure 4.**
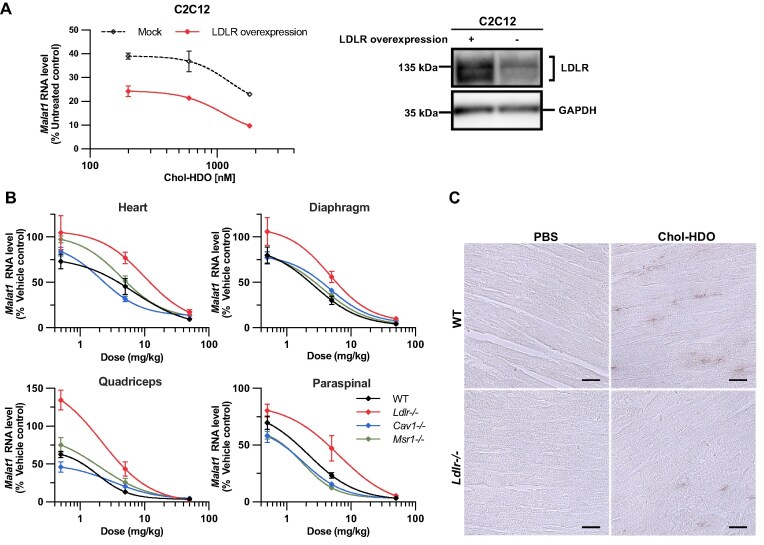
Dose–response curve of Chol-HDO in C2C12 cells overexpressing LDLR and in *Ldlr^−/−^, Cav1^−/−^*, and *Msr1^−/−^* mice. **(A)** Concentration–response curve of Chol-HDO (200, 600, and 1800 nM) in C2C12 cells with or without LDLR overexpression (left). Mature (∼160 kDa) and precursor LDLR (∼120 kDa) were detected in C2C12 cells transfected with an *Ldlr* plasmid or mock plasmid by western blot analysis (right). **(B)** Dose–response curve of Chol-HDO in WT, *Ldlr^−/−^, Msr1^−/−^*, and *Cav1^−/−^* mice following a single intravenous injection of Chol-HDO. *n* = 4–8 per PBS (vehicle control) group, *n* = 3–4 per Chol-HDO-treated group. **(C)** Immunohistochemical staining using an anti-PS antibody in the heart tissue harvested from WT or *Ldlr^−^*^/^*^−^* mice 3 days following an intravenous injection of 5 mg/kg of Chol-HDO. Scale bar, 20 μm. The doses represent the mass of the unconjugated ASO moiety. All the graphs show the data as mean ± SEM.

Because Chol-HDO exhibited high affinity for LDL (Fig. 3A), we hypothesized that Chol-HDO is taken up through a lipoprotein-mediated interaction with LDLR. To test this hypothesis, LDLR was overexpressed in C2C12 cells via transfection with a mouse *Ldlr*-expressing plasmid for 48 h. The cells were then treated with Chol-HDO in the presence of mouse serum. Chol-HDO exhibited a greater knockdown effect in LDLR-overexpressing cells compared with that in cells without overexpression (Fig. 4A). These results suggest that LDLR contributes to the efficient uptake of Chol-HDO.

### Chol-HDO activity in the cardiac and skeletal muscles of *Ldlr^−/−^, Cav1^−/−^*, and *Msr1^−/−^* mice

To identify the mechanism of action of Chol-HDO in muscle tissue, we employed three types of knockout mouse models: LDL receptor knockout (*Ldlr^−/−^*), Caveolin-1 knockout (*Cav1^−/−^*), and macrophage scavenger receptor 1 knockout (*Msr1^−/−^*). The mice received single IV injections of Chol-HDO targeting *Malat1* at the equivalent molar concentration of 0.5, 5, and 50 mg/kg of ASO. In the *Ldlr ^−/−^* mice, the ED_50_ for Chol-HDO was markedly increased in the cardiac and skeletal muscles compared with that in WT mice: 13.6 mg/kg versus 3.19 mg/kg in the heart, 6.33 mg/kg versus 2.09 mg/kg in the diaphragm, 4.84 mg/kg versus 0.821 mg/kg in the quadriceps, and 3.74 mg/kg versus 1.29 mg/kg in the paraspinal muscle (Fig. 4B). PS staining revealed decreased signals in the heart of *Ldlr*^−/−^ mouse relative to the WT mice (Fig. 4C). These results are consistent with the *in vitro* results showing that LDLR is involved in Chol-HDO activity (Fig. 4A). Conversely, in *Cav1^−/−^* and *Msr1^−/−^* mice, Chol-HDO showed comparable gene knockdown effects in both cardiac and skeletal muscles compared with that in the WT mice (Fig. 4B). These data suggest that LDLR, but not CAV1 or MSR1, is associated with the activity of Chol-HDO.

### Subcellular distribution of Chol-ASO and Chol-HDO

**Figure 5.**
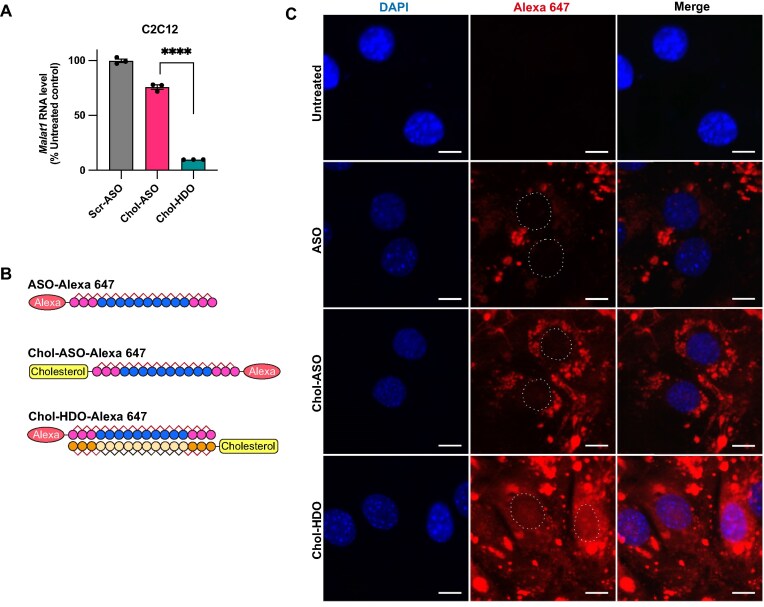
*In vitro* activity and subcellular distribution of unconjugated ASO, Chol-ASO, and Chol-HDO. **(A)** Knockdown activity of scrambled ASO, Chol-ASO, and Chol-HDO following gymnosis at 1800 nM with 1% mouse serum in C2C12 cells overexpressing LDLR. **(B)** Schematic representation of Alexa 647-labeled ASO, Chol-ASO, and Chol-HDO. **(C)** Subcellular localization of Chol-ASO and Chol-HDO following gymnosis with unconjugated ASO, Chol-ASO, or Chol-HDO at 400 nM in C2C12 cells overexpressing LDLR. Nuclei are indicated by circles. Scale bar, 10 μm. Error bars represent the SEM. Statistical analysis using Student’s two-tailed *t*-test was performed. ****P <.0001.

Chol-HDO exhibited superior knockdown activity compared with Chol-ASO *in vivo* (Fig. 1C). Consistently, when Chol-HDO and Chol-ASO were transfected into LDLR-overexpressing C2C12 cells, Chol-HDO showed greater potency compared with Chol-ASO (Fig. 5A), although the molecules share cholesterol conjugation. To identify the underlying mechanism for the greater potency of Chol-HDO, we assessed the subcellular distribution by measuring fluorescence intensity 24 h after gymnosis of Alexa 647-labeled unconjugated ASO, Chol-ASO, or Chol-HDO in C2C12 cells (Fig. 5B). The fluorescent signals were abundantly detected in the cytosol of cells treated with unconjugated ASO, Chol-ASO, and Chol-HDO; however, the cells treated with Chol-HDO exhibited stronger nuclear signals compared with those treated with unconjugated ASO and Chol-ASO (Fig. 5C). This suggests that the structure of Chol-HDO facilitates more efficient endosomal escape and nuclear delivery, which is consistent with its efficient knockdown activity (Figs 1C and 5A).

### Chol-HDO mitigates platelet activation caused by Chol-ASO

**Figure 6.**
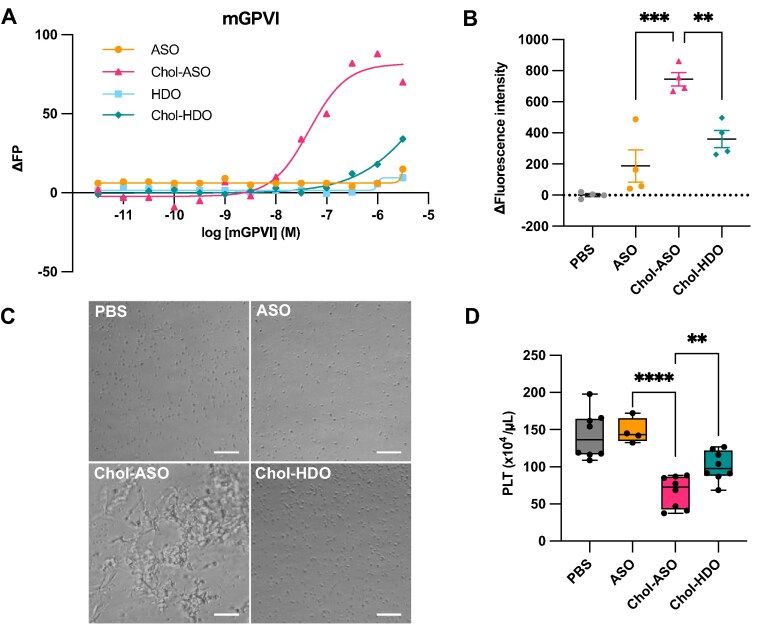
Platelet activation by Chol-ASO and Chol-HDO. **(A)** Binding curves of oligonucleotides to mouse glycoprotein VI (mGPVI), as assessed by FP. **(B)** Δfluorescence intensity of Fluo-8, a surrogate marker of calcium flux, in purified platelets treated with PBS or 12.5 µM of each oligonucleotide. The intensity of each sample was subtracted by the mean of the PBS control to calculate Δfluorescence intensity. **(C)** Phase-contrast microscopy images of mouse platelet-rich plasma treated with PBS or 12.5 µM of each oligonucleotide. Scale bar: 25 µm. **(D)** Platelet counts in mice 3 days following an intravenous injection of PBS or 25 mg/kg of each oligonucleotide. The doses represent the mass of the unconjugated ASO moiety. Data are presented as mean ± SEM. Statistical analysis using one-way analysis of variance followed by Dunnett’s test was performed. **P <.01; ***P <.001; ****P <.0001.

We examined the mechanism underlying the favorable tolerability of Chol-HDO. Toxicity remains a major limitation to the clinical application of ASO drugs. Although the PS-backbone facilitates protein binding and enhances cellular uptake, it also causes nonspecific protein binding and is associated with adverse events. For example, PS-ASOs activate platelets through the GPVI pathway [23–25], leading to platelet aggregation [43] and subsequent thrombocytopenia. Previous studies demonstrated that cholesterol conjugation to single-stranded ASO exacerbates this effect [24]. Moreover, we found that all mice treated with Chol-ASO targeting *Dmpk* exhibited pulmonary hemorrhage and lethality (Supplementary Fig. S5). To evaluate the effect of Chol-HDO on this pathway, we conducted an FP analysis to compare the binding affinity of *Malat1*-targeting unconjugated ASO, unconjugated HDO, Chol-ASO and Chol-HDO to GPVI. Chol-HDO exhibited weaker binding affinity to GPVI compared with Chol-ASO (Fig. 6A).

Next, we measured calcium mobilization as a surrogate marker for platelet activation. Purified platelets were incubated with Fluo-8 AM, a fluorescent calcium indicator. Following the addition of unconjugated ASO, Chol-ASO, or Chol-HDO, platelet activation was assessed by monitoring changes in fluorescent intensity. Chol-ASO induced a marked increase in intracellular calcium levels, whereas Chol-HDO exhibited a markedly attenuated response (Fig. 6B).

To further examine the direct effects on platelets, 25 µM of ASO, Chol-ASO, or Chol-HDO were added to mouse PRP. Consistent with a previous report [24], Chol-ASO induced platelet aggregation (Fig. 6C). In contrast, no aggregation was observed following treatment with either unconjugated ASO or Chol-HDO.

Finally, to determine the *in vivo* effects of Chol-ASO and Chol-HDO on platelets, mice were sacrificed 3 days after an IV injection of 25 mg/kg. Chol-ASO caused a significant reduction in platelet count compared with unconjugated ASO, whereas this effect was attenuated by Chol-HDO (Fig. 6D). These results indicate that, at an equivalent concentration, Chol-HDO reduces platelet activation, thereby mitigating thrombocytopenia.

## Discussion

In the present study, we demonstrated that Chol-HDO exhibits superior knockdown activity in muscle tissues compared with Chol-ASO and unconjugated ASO. FP analysis revealed that Chol-HDO significantly increased the binding affinity to LDL while minimizing the nonspecific protein interactions observed with Chol-ASO. Mechanistically, the Chol-HDO activity was associated with LDLR, which likely resulted from its efficient endothelial transcytosis and uptake by myocytes. Subcellular distribution analysis revealed that Chol-HDO, but not Chol-ASO, achieved efficient nuclear localization. Moreover, Chol-HDO induced less platelet activation and reduced the risk of thrombocytopenia compared with Chol-ASO at doses required for effective gene suppression in muscle tissue.

To assess the generalizability of the knockdown effect, we examined the knockdown of *Malat1, Dmpk*, and *Scarb1*. In all cases, Chol-HDO achieved greater gene suppression than unconjugated ASO. Although the molecular weight of HDO is approximately double that of ASO, resulting in an approximate twofold higher weight-based dose for Chol-HDO, its potency (ED_90_ or ED_50_) was improved by 2.7- to 9.5-fold, indicating superior potency per weight-based dose. Furthermore, Chol-HDO mitigated thrombocytopenia associated with high-dose Chol-ASO. Therefore, Chol-HDO represents an attractive modality in terms of both efficacy and safety, despite its increased weight-based dose.

The binding affinity of unconjugated HDO for serum proteins was lower than that of ASO, which is consistent with a previous report [36, 44]. The duplex structure of HDO reduces molecular flexibility [36] and hides sulfur atoms, which are major sites for ASO–protein interactions [44–46], in its major groove [47]. These factors may limit its nonspecific protein interactions. In contrast, cholesterol conjugation enhanced the binding affinity of both ASO and HDO for LDL. However, while Chol-ASO also exhibited higher affinity for albumin, IgG, Tf, and GPVI than ASO, Chol-HDO exhibited comparable affinities to these proteins. These observations suggest that the structural rigidity of HDO effectively minimizes nonspecific protein interactions, whereas cholesterol conjugation enables selective binding to physiologically relevant lipoproteins. The decreased nonspecific binding of Chol-HDO, including its binding to GPVI may attenuate platelet activation compared with Chol-ASO, thereby lowering the risk of thrombocytopenia. This is supported by the previous reports demonstrating the favorable safety profile of duplex oligonucleotides [47, 48].

LDLR is a cell-surface receptor that mediates the endocytosis of LDL, intermediate-density lipoprotein, very-low-density lipoprotein, and high-density lipoprotein. It is ubiquitously expressed in endothelial cells and cardiac and skeletal muscles [49–51]. We found that LDLR plays an important role in the activity of Chol-HDO *in vitro*. A dose–response study employing LDL receptor knockout mice (*Ldlr*^−/−^) [52] revealed that the knockdown activity of Chol-HDO in the cardiac and skeletal muscles was significantly decreased in *Ldlr^−/−^* mice compared with WT mice. Consistently, the PS signal was also reduced in the muscle tissue of *Ldlr^−/−^* mice. Because LDLR is expressed in both endothelial cells and myocytes, it likely mediates endothelial transcytosis and cellular uptake from the interstitial space.

Caveolin-mediated transcytosis is a clathrin-independent, dynamin-dependent, endocytic process involving bulb-shaped plasma membrane invaginations known as caveolae. Although muscular cells lack Cav1 expression, endothelial cells express Cav1. Serum albumin, immunoglobulins, and lipoproteins are transported across the endothelium into the interstitial space through caveolin1-mediated transcytosis [50, 53]. The activity of Palm-ASO was previously shown to be reduced in the heart and quadriceps of *Cav1^−/−^* mice, likely because albumin-bound Palm-ASO are transported across endothelial cells via Cav1 [16]. However, we found that the potency of Chol-HDO in *Cav1^−/−^* mice was similar to that in WT mice. Because Chol-HDO exhibits substantially higher affinity for LDL, but not for albumin, we speculated that endothelial transcytosis of Chol-HDO was primarily mediated by the LDL–LDLR axis.

We further examined MSR1 as a possible candidate for the Chol-HDO delivery pathway. MSR1 is a functional receptor that mediates endocytosis of the modified LDL [54]. A previous study reported that the cellular uptake of ASO and Pip6a-morpholino oligomer is associated with MSR1 [8, 55, 56]. In addition, we previously demonstrated that activated microglia and macrophages in the central nervous system of animals with experimental autoimmune encephalomyelitis take up Chol-HDO via MSR1 [31]. To elucidate the involvement of MSR1 in the delivery of Chol-HDO to the cardiac and skeletal muscles, we examined the gene knockdown effect of Chol-HDO in *Msr1^−/−^* mice. Unexpectedly, the knockdown effect was unchanged in cardiac and skeletal muscles of *Msr1^−/−^* mice. These results indicate that MSR1 is unlikely to function as a major receptor mediating the transport of Chol-HDO, although the potential compensatory upregulation of other scavenger receptors cannot be excluded.

Following endocytic internalization, oligonucleotides are transported through endosomes, and their escape from these compartments is an important step for achieving therapeutic efficacy [41, 57]. Most single-stranded ASOs are reported to be entrapped in endosomes and degraded in lysosomes [58, 59]. In contrast, we previously found that ASO is released from HDO in early endosomes and is rapidly transported to the nucleus following lipofection of unconjugated HDO [60]. Consistent with this result, we observed that Chol-HDO was more efficiently delivered to the nucleus than Chol-ASO after gymnosis in differentiated C2C12 cells. This efficient endosomal escape and nuclear distribution may contribute to the higher activity of Chol-HDO compared with Chol-ASO. Endosomal release of PS-ASOs has been reported to be mediated by back-fusion, membrane leakage, and vesicle-mediated pathways [41, 61, 62]. Although the precise mechanism remains unclear, Chol-HDO’s intracellular protein-binding profile may (i) facilitate interaction with proteins that promote productive trafficking and nuclear access and/or (ii) reduce interactions that otherwise sequester PS-ASOs in nonproductive compartments, thereby increasing the fraction that reaches the nucleus.

In conclusion, systemically injected Chol-HDO exhibits more efficient knockdown of a target gene in cardiac and skeletal muscles than unconjugated ASO or Chol-ASO. Furthermore, Chol-HDO attenuates thrombocytopenia induced by Chol-ASO. Further comprehensive screening for optimal ligands targeting the muscle tissue may improve the efficacy and safety of HDO. Although further evaluations in nonhuman primates are essential to assess the clinical applicability of Chol-HDO, our study observed that Chol-HDO holds substantial potential as a therapeutic option for treating muscular disorders.

## Supplementary Material

gkag007_Supplemental_File

## Data Availability

The data associated with this article are available in the article and online supplementary material.
