# Supplementary material for: Heteroduplex oligonucleotide technology boosts gene knockdown in cardiac and skeletal muscles
Source: Nucleic Acids Res. 2026 Jan 28;54(3):gkag007. doi: 10.1093/nar/gkag007 (PMC12846741; doi:10.1093/nar/gkag007)
Supplement: gkag007_Supplemental_File [file gkag007_supplemental_file.pdf]

SUPPLEMENTARY DATA

Supplementary Table S1. Summary of ASOs and HDOs

ASO<br>(*Malat1*)	5′-CTAGTTCACTGAATGC-3′	ss
5′ Chol-ASO<br>(*Malat1*)	5′-Chol-CTAGTTCACTGAATGC-3′	ss
HDO<br>(*Malat1*)	5′-CTAGTTCACTGAATGC-3′<br>3′-GAU*CAAGUGACUU*ACG-5′	ds
Toc-HDO<br>(*Malat1*)	5′-CTAGTTCACTGAATGC-3′<br>3′-GAU*CAAGUGACUU*ACG-Toc-5′	ds
Palm-HDO<br>(*Malat1*)	5′-CTAGTTCACTGAATGC-3′<br>3′-GAU*CAAGUGACUU*ACG-Palm-5′	ds
5′ Chol-HDO (Chol-HDO)<br>(*Malat1*)	5′-CTAGTTCACTGAATGC-3′<br>3′-GAU*CAAGUGACUU*ACG-Chol-5′	ds
3′ Chol-HDO<br>(*Malat1*)	5′-CTAGTTCACTGAATGC-3′<br>3′-Chol-GAU*CAAGUGACUU*ACG-5′	ds
Scr-*Malat1* ASO<br>(*Malat1* Scrambled)	5′- ACGTGATCGCCTTATA -3′	ss
Scr-*Malat1* Chol-HDO<br>(*Malat1* Scrambled)	5′- ACGTGATCGCCTTATA -3′<br>3′- UGC*ACUAGCGGAA*UAU -Chol-5′	ds
ASO<br>(*Dmpk*)	5′-ACAATAAATACCGAGG-3′	ss
Toc-HDO<br>(*Dmpk*)	5′-ACAATAAATACCGAGG-3′<br>3′-UGU*UAUUUAUGGC*UCC-Toc-5′	ds

Chol-HDO<br>(*Dmpk*)	5′-ACAATAAATACCGAGG-3′<br>3′-UGU*UAUUUAUGGC*UCC-Chol-5′	ds
Palm-ASO<br>(*Dmpk*)	5′-Palm-TCAACAATAAATACCGAGG-3′	ss
Chol-ASO<br>(*Dmpk*)	5′-Chol-ACAATAAATACCGAGG-3′	ss
Scr-*Dmpk* ASO<br>(*Dmpk* Scrambled)	5′- AGACGCTATAAACGAA -3′	ss
Scr-*Dmpk* Chol-HDO<br>(*Dmpk* Scrambled)	5′- AGACGCTATAAACGAA -3′<br>3′- UCU*GCGAUAUUUG*CUU-Chol-5′	ds
ASO<br>(*Scarb1*)	5′-TCAGTCATGACTTC-3′	ss
Toc-HDO<br>(*Scarb1*)	5′- TCAGTCATGACTTC -3′<br>3′- AG*UCAGUACUGA*AG-Toc-5′	ds
Chol-HDO<br>(*Scarb1*)	5′- TCAGTCATGACTTC -3′<br>3′- AG*UCAGUACUGA*AG -Chol-5′	ds

Note: The orange font indicates locked nucleic acids (LNAs); the red font indicates 2′-*O*-methyl (OMe); the black font denotes DNA; italicized letters represent ribonucleic acid (RNA); the phosphorothioate (PS) backbone, unless underlined, indicates a phosphodiester (PO) linkage; A, adenine; ASO, antisense oligonucleotide; C in the wing portion of ASO, 5-methylcytosine; Chol, cholesterol; DNA, deoxyribonucleic acid; G, guanine; HDO, heteroduplex oligonucleotide; Palm, palmitic acid; RNA, ribonucleic acid; ss, single strand; ds, double strand; T, thymine; Toc, tocopherol; U, uracil.

Supplementary Table S2. Primers and probes used in SplintR qPCR

Dmpk ASO primer A	5′-CTCGACCTCTCTATGGGCAGTCACGACAGCCTCGGTA-3′
Dmpk ASO primer B	5′-pTTTATTGTCGCTGAGTCGGAGACACGCAGGGCTTAA-3′
Forward primer	5’-GCTCGACCTCTCTATGGGC-3′
Reverse primer	5’-TTAAGCCCTGCGTGTCTCC-3’
Double-quenched probe	5’-/FAM/TAAATACCG/ZEN/AGGCTGTCGTG/IABkFQ/-3’

Note: p, 5’ phosphate; FAM, 6-carboxyfluorescein; ZEN, internal quencher; IABkFQ, Iowa Black FQ.

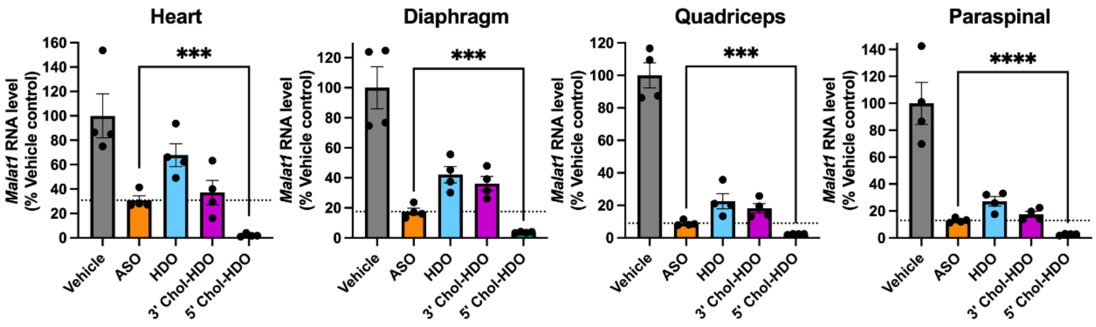

Supplementary Figure S1. Knockdown activity of ASO, unconjugated HDO, 3′ Chol-HDO, and 5′ Chol-HDO

In vivo gene silencing of *Malat1* in the heart, diaphragm, quadriceps, and paraspinal muscles, 3 days following a single intravenous injection of each oligonucleotide at a molar dose equivalent to 50 mg/kg unconjugated ASO (n = 4 per group). The data are presented as mean ± SEM. Statistical significance was assessed using Student's t-test comparing ASO and 5′ Chol-HDO. ***P < 0.001; ****P < 0.0001.

A

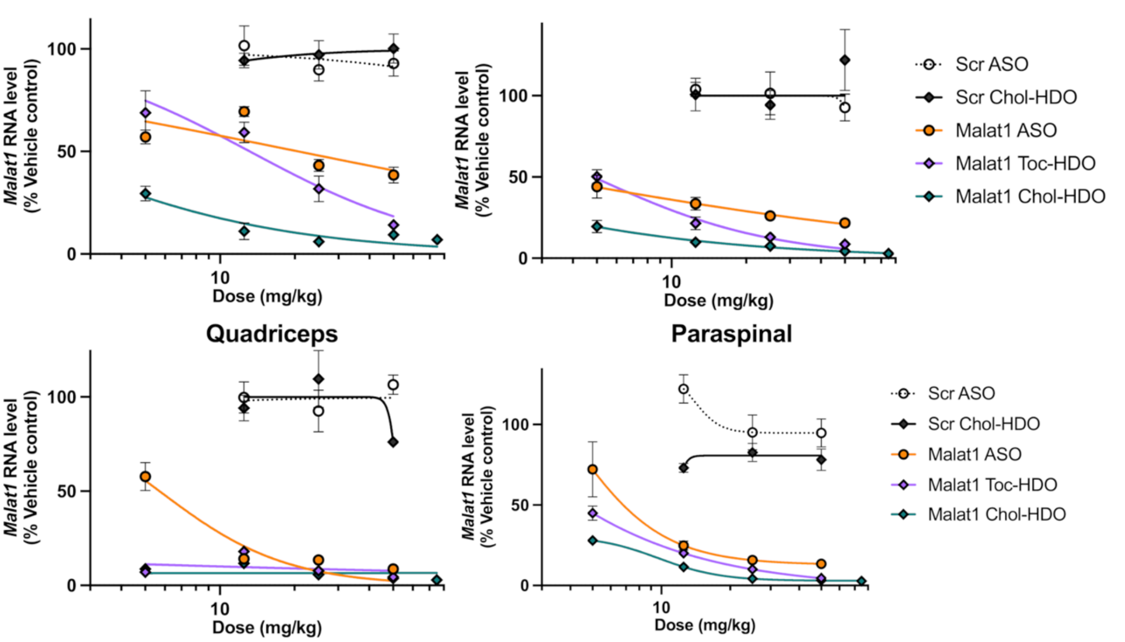

B

		ED90 (mg/kg single IV)			
		Heart	Diaphragm	Quadriceps	Paraspinal
Malat1	ASO	> 50.0	> 50.0	40.5	> 50.0
	Toc-HDO	> 50.0	38.8	7.45	32.2
	Chol-HDO	18.3	11.9	6.59	16.3

Supplementary Figure S2. Dose–response curve of Malat1 RNA knockdown

(A) Dose–response curves showing *Malat1* knockdown in the heart, diaphragm, quadriceps, and paraspinal muscles following a single intravenous injection of *Malat1* unconjugated ASO, Toc-HDO, or Chol-HDO targeting *Malat1*, scrambled (Scr) ASO, or scrambled Chol-HDO (n = 4 per group). Tissues were harvested 3 days after injection. The data are presented as mean ± SEM. (B) ED_{90} values (mg/kg) for *Malat1* knockdown in each tissue following the administration of ASO, Toc-HDO, or Chol-HDO. The doses represent the mass of the unconjugated ASO moiety.

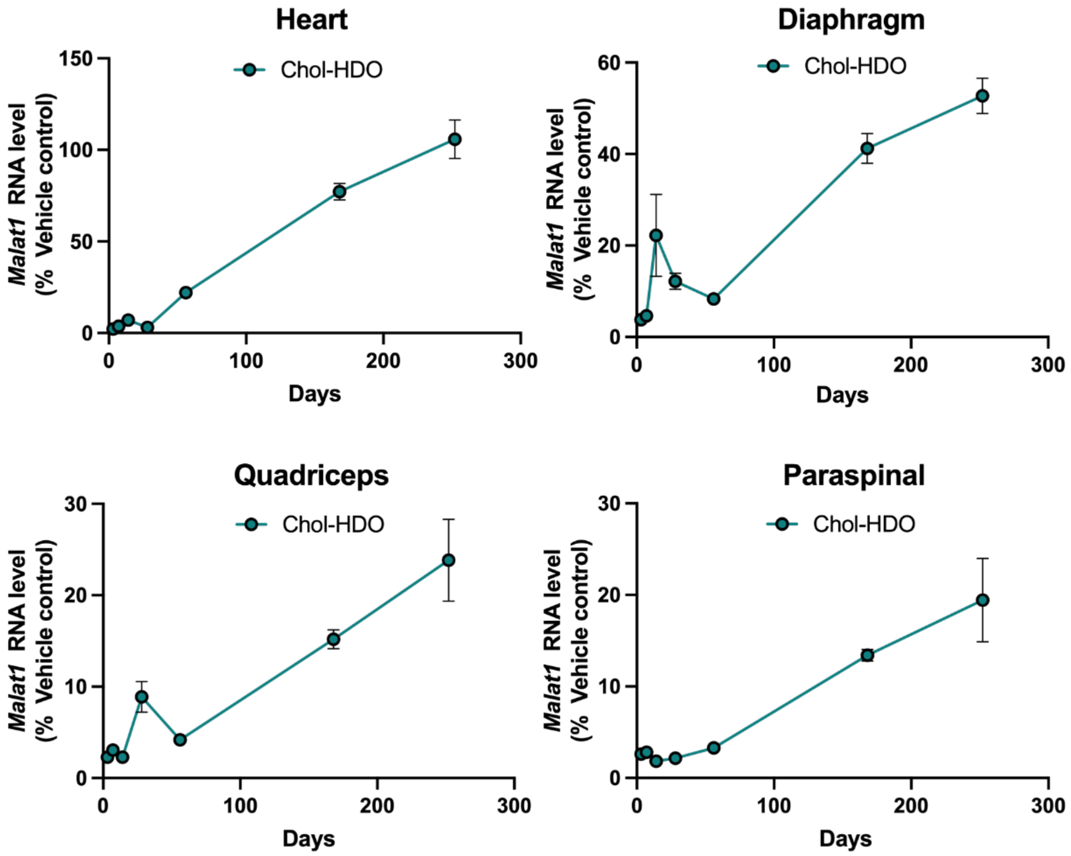

Supplementary Figure S3. Time course of RNA reduction after a single administration in the muscles

(A) Time course of *Malat1* RNA reduction in the heart, diaphragm, quadriceps, or paraspinal muscles on days 3, 7, 14, 28, 56, 168, or 252 following a single intravenous injection of Chol-HDO at a molar equivalent dose of 50 mg/kg of ASO (n = 4 per group). All data are presented as the mean ± SEM.

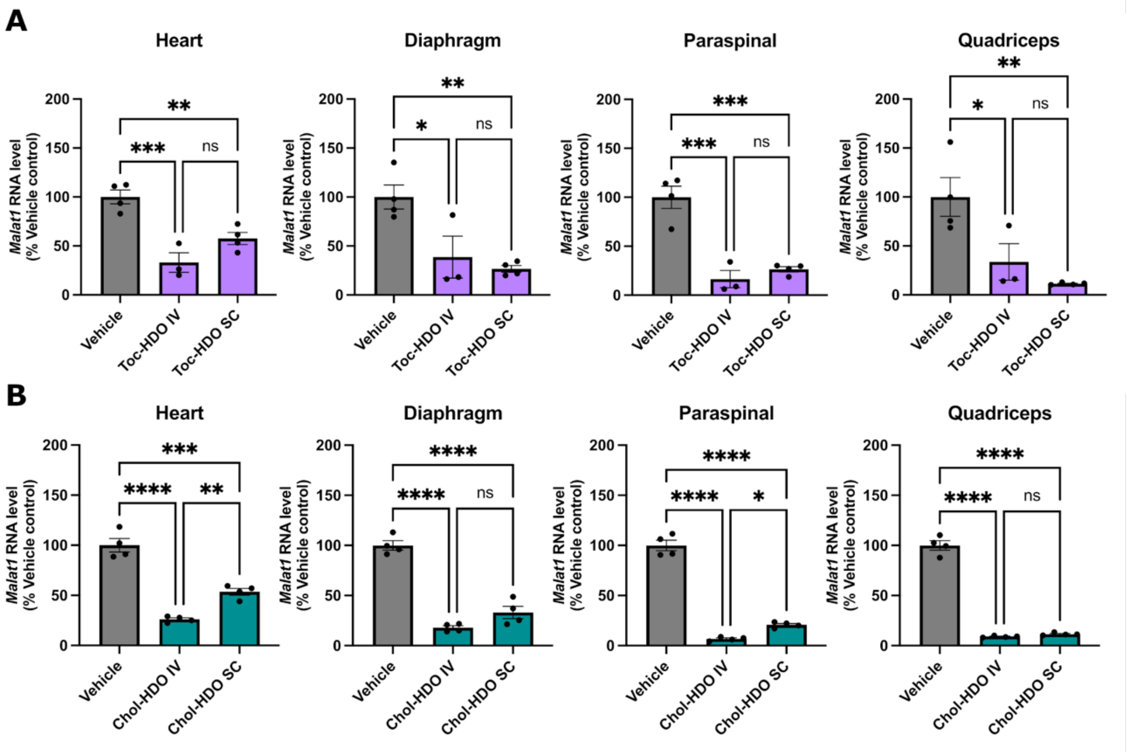

Supplementary Figure S4. Gene silencing efficacy of Toc-HDO or Chol-HDO targeting *Malat1* in the muscles following a single intravenous or subcutaneous injection

(**A**) The effect of the administration route of Toc-HDO. *Malat1* RNA levels in the heart and skeletal muscles on day 7 following a single intravenous or subcutaneous injection of Toc-HDO at a dose of 25 mg/kg. *n* = 3–4 per group. (**B**) The effect of the administration route of Chol-HDO. *Malat1* RNA levels in the heart and skeletal muscles on day 7 following a single intravenous or subcutaneous injection of Chol-HDO at a dose of 12.5 mg/kg. *n* = 4 per group. Doses represent the mass of the unconjugated ASO moiety. All data are presented as mean ± SEM. Statistical analysis was performed using one-way analysis of variance followed by Tukey's multiple comparisons test. *P < 0.05; **P < 0.01; ***P < 0.001; ****P < 0.0001; ns, not significant.

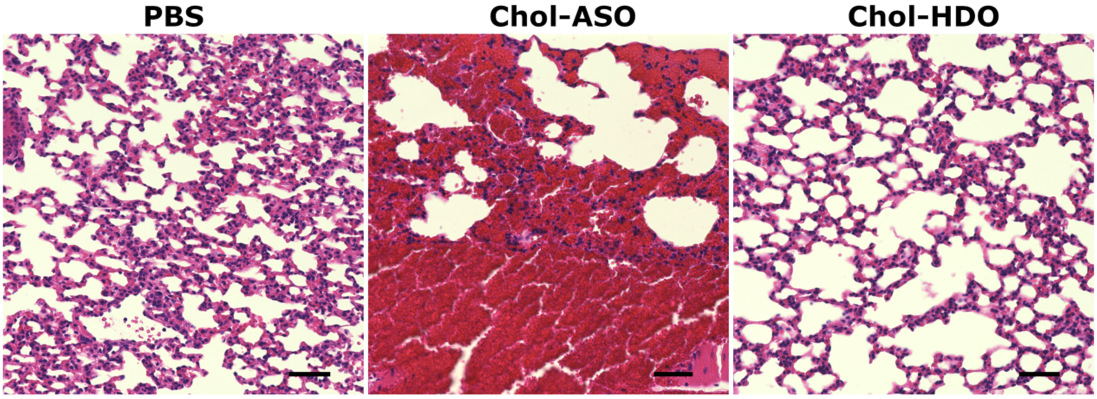

Supplementary Figure S5. Alveolar hemorrhage in the lungs of mice treated with Chol-ASO targeting *Dmpk*

Intravenous injection of 25 mg/kg Chol-ASO targeting *Dmpk* was lethal within 24 h. An autopsy revealed massive pulmonary alveolar hemorrhage in all Chol-ASO–treated mice, whereas no alveolar hemorrhage was observed in the PBS- or Chol-HDO–treated groups at the same dose (n = 4 per group). Hematoxylin–eosin staining. Scale bar, 50 μm. Doses represent the mass of the unconjugated ASO moiety.

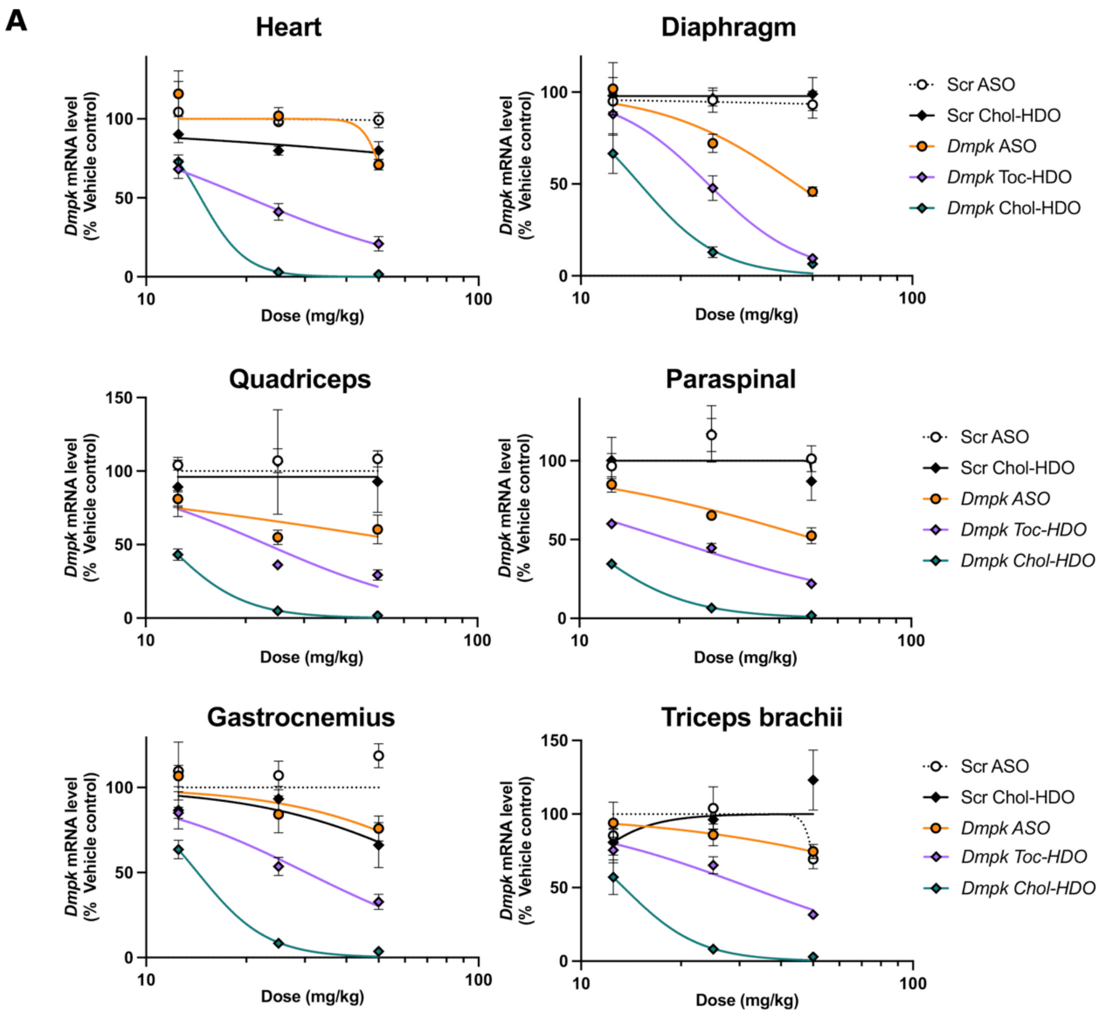

B

Dmpk		ED50 (mg/kg single IV)					
		Heart	Diaphragm	Quadriceps	Paraspinal	Gastrocnemius	Triceps brachii
	ASO	> 50.0	44.7	> 50.0	> 50.0	> 50.0	> 50.0
	Toc-HDO	20.3	24.2	23.2	18.8	30.3	32.4
	Chol-HDO	14.6	15.1	11.6	10.0	14.3	13.5

Supplementary Figure S6. The dose–response curve obtained after a single intravenous administration of ASO, Toc-HDO, or Chol-HDO targeting *Dmpk* in the heart and skeletal muscles of mice

(A) Dose–response curve showing *Dmpk* knockdown in the heart, diaphragm, quadriceps, paraspinal muscles, gastrocnemius, or triceps brachii following a single intravenous injection of PBS (vehicle control); *Dmpk*-targeting unconjugated ASO, Toc-HDO, or Chol-HDO; and scrambled ASO. $n = 4$ per group. The muscles were harvested 3 days after dosing. All data are presented as mean ± SEM.

(B) ED_{50} (mg/kg) for reducing *Dmpk* mRNA levels in the heart, diaphragm, quadriceps, paraspinal muscles, gastrocnemius, or triceps brachii by unconjugated ASO, Toc-HDO, or Chol-HDO. The doses represent the mass of the unconjugated ASO moiety.

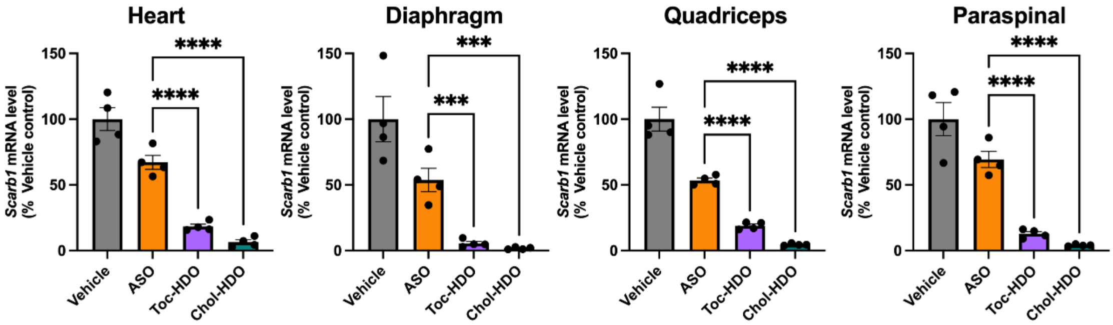

Supplementary Figure S7: Gene silencing activity of Toc-HDO or Chol-HDO targeting *Scarb1* in the muscles after systemic injection

In vivo knockdown of *Scarb1* in the heart, diaphragm, quadriceps, or paraspinal muscles following a single IV injection of PBS (vehicle control), 50 mg/kg of unconjugated ASO, and equimolar doses of Toc-HDO or Chol-HDO. The muscles were harvested 3 days after dosing ($n = 4$ per group). The data are presented as mean ± SEM. Statistical analysis was performed using one-way analysis of variance followed by Dunnett's test. *P < 0.05; **P < 0.01; ***P < 0.001; ****P < 0.0001; ns: not significant.

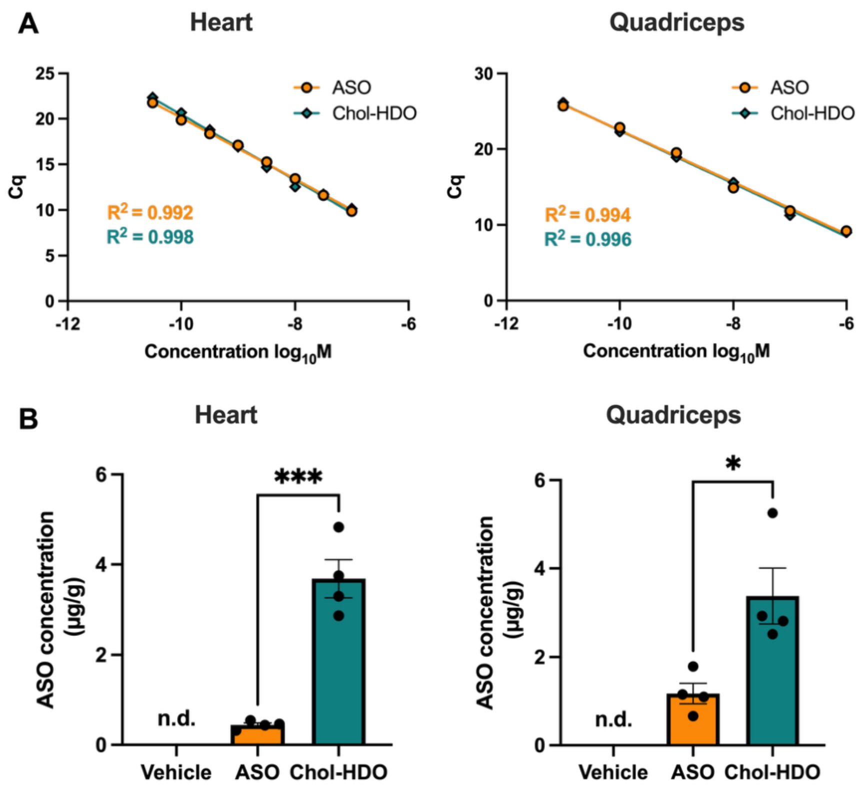

Supplementary Figure S8. ASO concentrations in muscle tissues from mice treated with ASO or Chol-HDO quantified by SplintR qPCR.

(A) Standard curves generated by spiking the indicated amounts of ASO or Chol-HDO into mouse heart or quadriceps tissue. The standard curves for ASO and Chol-HDO were comparable. Cq, quantification cycle. (B) ASO concentrations in the heart and quadriceps three days after a single IV injection of 12.5 mg/kg unconjugated ASO targeting *Dmpk*, an equimolar dose of Chol-HDO, or PBS (vehicle control) (n = 4 per group). Data are presented as mean ± SEM. Statistical significance was assessed using Student's *t*-test. n.d., not detected (below the detection limit of the assay); *P < 0.05; ***P < 0.001.

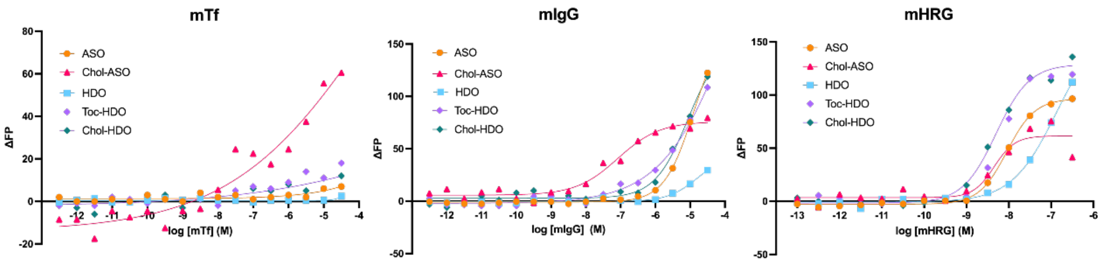

Supplementary Figure S9. The interaction of oligonucleotides with mouse transferrin, immunoglobulin G, or histidine-rich glycoprotein

Binding curves of unconjugated ASO, Chol-ASO, unconjugated HDO, Toc-HDO, and Chol-HDO to mouse transferrin (mTf), mouse immunoglobulin G (mIgG), or mouse histidine-rich glycoprotein (mHRG), as measured using a fluorescence polarization assay.
